# Characterization of Coal Porosity for Naturally Tectonically Stressed Coals in Huaibei Coal Field, China

**DOI:** 10.1155/2014/560450

**Published:** 2014-07-10

**Authors:** Xiaoshi Li, Yiwen Ju, Quanlin Hou, Zhuo Li, Mingming Wei, Junjia Fan

**Affiliations:** ^1^Key Laboratory of Computational Geodynamics, Chinese Academy of Sciences, College of Earth Science, University of Chinese Academy of Sciences, Beijing 100049, China; ^2^State Key Laboratory of Petroleum Resource and Prospecting, China University of Petroleum, Beijing 102249, China; ^3^PetroChina Research Institute of Petroleum Exploration & Development, Key Lab of Basin Structure and Petroleum Accumulation, Beijing 100083, China

## Abstract

The enrichment of coalbed methane (CBM) and the outburst of gas in a coal mine are closely related to the nanopore structure of coal. The evolutionary characteristics of 12 coal nanopore structures under different natural deformational mechanisms (brittle and ductile deformation) are studied using a scanning electron microscope (SEM) and low-temperature nitrogen adsorption. The results indicate that there are mainly submicropores (2~5 nm) and supermicropores (<2 nm) in ductile deformed coal and mesopores (10~100 nm) and micropores (5~10 nm) in brittle deformed coal. The cumulative pore volume (*V*) and surface area (*S*) in brittle deformed coal are smaller than those in ductile deformed coal which indicates more adsorption space for gas. The coal with the smaller pores exhibits a large surface area, and coal with the larger pores exhibits a large volume for a given pore volume. We also found that the relationship between *S* and *V* turns from a positive correlation to a negative correlation when *S* > 4 m^2^/g, with pore sizes <5 nm in ductile deformed coal. The nanopore structure (<100 nm) and its distribution could be affected by macromolecular structure in two ways. Interconversion will occur among the different size nanopores especially in ductile deformed coal.

## 1. Introduction

Deformation and metamorphism could occur in coal to different extents under stable pressure conditions after coal is formed. As a complex organic material with a complicated porous structure, coal has been studied by many researchers on the relationships between the macromolecular structure and the nanopore structure [1–3]. Duber and Rouzaud discussed the change between the pores and the macromolecular structure of coal and proposed that the size and shape of the pores in coal depend on coalification, the temperature, and the anisotropic stress, which can affect the metamorphic grade of coal [1]. Cao et al. considered that tectonic stress could affect the chemical structure of coal, thereby leading to changes in the macromolecular structure, which could affect the nanopore structure (<100 nm) and its distribution [2–4].

The nanopore structure of coal is the most important reservoir space for gas. Some scholars indicate that the pore (2~5 nm) is the primary factor that controls the gas adsorption capacity [5, 6]. Various studies have identified the most important factors influencing the pore properties (structure and distribution) and include coal rank (*R*_*o*_) [7–9], maceral composition [8, 9], gas sorption [9, 10], and deformation [11–14]. Coal rank describes the degree of carbonification of the coal. Coal rank may be determined by a variety of physical and chemical properties, some of which are influenced by maceral composition [7]. Swelling induced in coal by gas sorption and strain is also associated with the changes of pore structure of coal [9, 10]. Xue et al. studied the coal deformed by tectonic stress and considered that tectonic deformation could reform the pores structure and connectivity of the pore network [11–14]. However, the deformed coal with different deformational mechanisms (brittle and ductile deformation) could also affect the pore structure of coal [2, 15], but the characteristics of the nanopore structure of coal under different tectonic deformations are rarely reported. Therefore, research on the evolutionary process of nanopore structure in coal under tectonic deformation, deformed coal, is discussed here. To minimise the effects of coal rank and maceral composition, we have chosen to use the medium rank coal samples and treated those coal samples with demineralization and vitrinite centrifugation processes to increase the vitrinite content [16]. The detailed characteristics of those two series coals will be described later.

Various methods such as scanning electron microscopy (SEM), transmission electron microscopy (TEM), mercury porosimetry, N_2_ adsorption at 77 K, and small-angle X-ray/neutron scattering (SAXS/SANS) have been used to study the pore structure [4, 17, 18] of coals. Nitrogen adsorption at boiling temperature (77 K) represents the most widely used technique to determine coal surface area and to characterize its porous structure [17]. The methods based on Density Functional Theory (DFT) represent the more recent approach to the calculation of pore volume and pore size distribution (particularly for micropores) from adsorption isotherm [17, 19]. All 12 deformed coal samples (medium rank) were collected from a Permian-Carboniferous coalbed in Huaibei coal field. We studied the data resulting from SEM images (magnification: 200–4000) and low-temperature nitrogen adsorption (DFT method), which exposes the nanopore structure to reveal the characteristics of coal porosity, formation processes, and mechanisms of nanopore structure in crushed deformed coal samples under different deformation-metamorphism environments.

## 2. Samples and Experiment Methods

### 2.1. Samples and Experimental Methods

The 12 deformed coal samples with different deformation and metamorphism characteristics were collected from a Permian-Carboniferous coalbed in Huaibei coal field, which was strongly affected by the Mesozoic tectonic deformation. The Huaibei coalfield is typically composed of various tectonically deformed coals containing rich coalbed methane resources. The coal seams are distributed mainly in graben part, especially in the syncline part distributed in north-south tectonic blocks and east-west tectonic zones [14, 16, 20, 21]. There are 11 block coal samples which were obtained from different coal seams in TaoYuan (Y, coal seams 8 and 10), LuLing (L, coal seams 8 and 10), LinHuan (H, coal seams 7, 9 and 10), ShiTai (T, coal seam 3), HaiZi (Z, coal seams 8 and 10) coal mines, and one core sample from TaoYuan (K) coal mine.

Coal rank (*R*_*o*_) data of all deformed coal samples were tested first on a ZEISS lmager M1m microspectrophotometer according to the standard GB/T6948-2008 at 23°C; the reflectance of immersion oil (*N*_*e*_) is 1.5180. More than 500 points were tested on each coal sample.

SEM images of 12 untreated samples were tested on JSM-6390LV scanning electron microscope (acceleration voltage: 0.5 kv~30 kv; beam: 1 pA~1 *μ*A) used for 2D pore topography analysis. Smooth particles were selected from the untreated block samples. Before SEM, the coal samples were polished and sputter-coated with a layer of gold. The micromorphology of different deformational mechanisms and surface configuration in deformed coal can be intuitively observed.

Before adsorption/desorption test, 12 deformed coal samples were treated with demineralization first, by reducing the proportion of mineral matter in each sample to less than 2% using hydrochloric acid (HCl) and hydrofluoric acid (HF). Then by vitrinite centrifugation, we use benzene and carbon tetrachloride (CCl_4_) to refine the amount of vitrinite up to 75%–99% [16]. Gürdal et al. find that the indicative correlation could not be observed between the pore properties and maceral composition, but the vitrinite content showed a weak correlation with the pore properties [8, 9]. Those processes will increase the vitrinite content and minimise the effects of maceral composition on pore structure. Low-temperature nitrogen adsorption on 12 treated samples was tested on a NOVA4200e pore specific surface area and a porosity analyzer under liquid nitrogen temperature (77 K). The minimum measurement of the specific surface area is 0.01 m^2^/g, and the measurement range of the pore size is from 3.5 to 2000 Å. Samples were dried in an oven for two hours at 60°C first and then vacuum-heated to remove the gas for 12 hours under 70°C before the test.

The results of vitrinite reflectance, maceral, and composition of deformed coal samples were shown in Table 1. The 12 deformed coal samples are medium rank coals, vitrinite reflectance between 0.83 and 1.93%.

Maceral analysis showed that the dominant composition are vitrinite with an average of 85.1% (between 77.9% and 94.09%) and inertinite with an average of 11.6% (2.50–18.37%), followed by liptinite with an average of 2.4%, and a little of mineral. Because of the treating processes (demineralization and vitrinite centrifugation), the content of liptinite, inertinite, and mineral is very low. Similar vitrinite content was shown in two series coals, respectively. In brittle deformed coals, vitrinite content ranges from 76.83 to 86.09% and 82.55 to 94.09% in ductile deformed coals.

### 2.2. Low-Temperature Nitrogen Adsorption Data Processing

Because of its high accuracy and reduced calculation load in describing a heterogeneous system, the Density Functional Theory (DFT) is widely used in the establishment of porous material models [19]. DFT is based on the principle of the molecular statistical theory and thermodynamic argument. DFT considers the adsorption between the mass and between the adsorbate and adsorbent, using an approach that is ultimately based on the principle of minimum potential energy of thermodynamics.

The isotherm determined using the DFT can be expressed mathematically by

(1) $$\begin{array}{c} Q\left(p\right)=\int q\left(p,H\right)f\left(H\right)dH, \end{array}$$

where *Q*(*p*) is the adsorbing capacity when the pressure is *p*; *q*(*p*, *H*) is the adsorbing capacity when the pore width is *H* under pressure *p*, and *f*(*H*) is the area that corresponds to the pore width *H*.

The data of the porosity and the pore size distribution could be calculated by the sample isotherm [22, 23]. The relevant parameters of a smaller pore structure can be measured by the DFT model, and the result of the smaller pore structure is much more accurate than that of the larger pore structure. For the smallest pore width of 1.03 nm, we compared the parameters of the pore structure under the different deformational mechanisms to study the evolutionary characteristics of the coal nanopore structure.

The adsorption parameters data of 12 deformed coal samples were listed in Table 2. The cumulative specific surface area and pore volume for ductile deformed coals range from 2.3708 to 4.1434 m^2^/g and 0.0092 to 0.012 cm^3^/g, respectively. Those parameters for brittle deformed coal samples are relatively small, ranging from 1.1800 to 2.5297 m^2^/g and 0.0047 to 0.0095 cm^3^/g, respectively. Most of the samples possess pore average width between 11 nm and 16 nm. The pore average width not only could be up to about 20 nm in brittle deformed coals (sample L01), but also could be smaller than 8 nm (samples L04 and K04) in ductile deformed coals.

### 2.3. Classification of the Nanopore Structure of Deformed Coal

There are many classifications of the nanopore structure; however, due to the influence of tectonic deformation, the conventional classification of the primary structure of coal is not suitable for deformed coal. In this paper, we used the combined classifications from IUPAC, Hodot, Ju et al., and Cai et al., which are suitable for research on the nanopore structure of deformed coal [6, 24–26] (Table 3).

## 3. Results and Discussions

### 3.1. Macro- and Microscopic Characteristics of Deformed Coal

**Figure 1:**
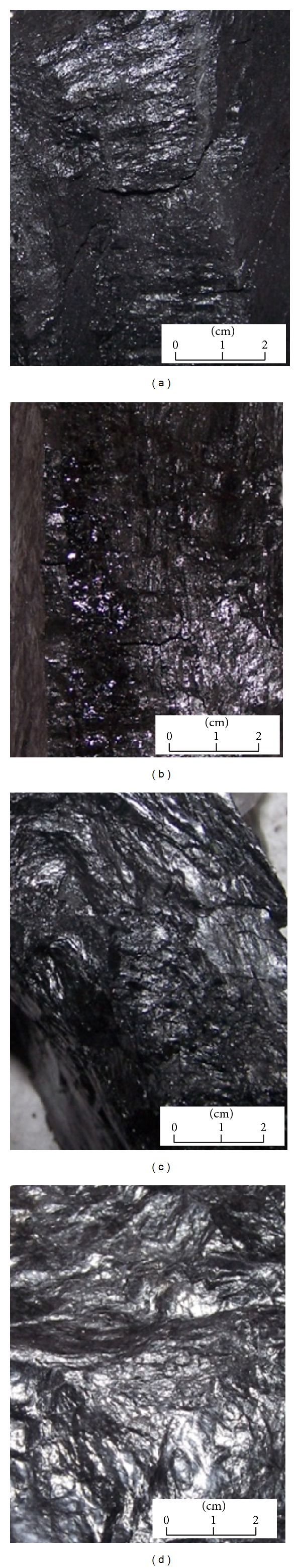
Hand specimen images of deformed coal, (a) weak brittle deformed coal (Y04); (b) strong brittle deformed coal (H12); (c) weak ductile deformed coal (L04); (d) strong ductile deformed coal (H09).

The samples were divided into two series: brittle deformation and ductile deformation [15]. The weak brittle deformed coal (Figure 1(a)) usually has multidirectional or unidirectional fractures, and the primary structure could be observed; the coal was hard and not easily broken. In strong brittle deformed coal (Figure 1(b)), the primary structure is damaged and the coal bedding has almost disappeared. Coal shows subangular or subround particles, has low strength, and could be disintegrated by hand.

However, the weak ductile deformed coal (Figure 1(c)) has a wrinkled or mylonitic structure, the maceral of coal not easily discriminated by the naked eye; the coal was soft and could be easily pinched into fragments. Under strong ductile deformation, the coal is strongly wrinkled and forms irregular crumb structures which cannot be discriminated. The coal could be turned into fine grains by hand (Figure 1(d)).

**Figure 2:**
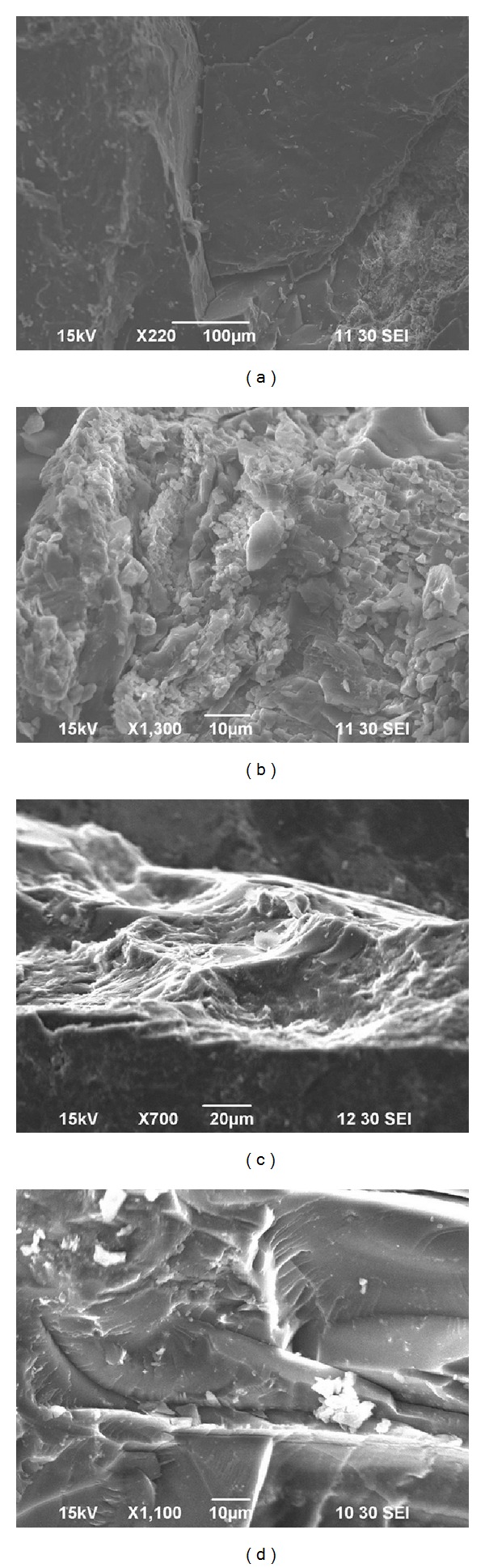
SEM images of deformed coal, (a) weak brittle deformed coal (Y04); (b) strong brittle deformed coal (H12); (c) weak ductile deformed coal (L04); (d) strong ductile deformed coal (H09).

Figure 2 show SEM images of the deformed coal samples. The micromorphology of different deformational mechanisms and the surface configuration in deformed coal were observed. Figures 2(a) and 2(b) show the microscopic characteristics of brittle deformed coal, which exhibits fractures, parallel bedding, and different grain sizes. Figures 2(c) and 2(d) show the microscopic characteristics of ductile deformed coal, which clearly exhibit a wrinkled structure.

### 3.2. N_2_ Gas Adsorption Isotherms

**Figure 3:**
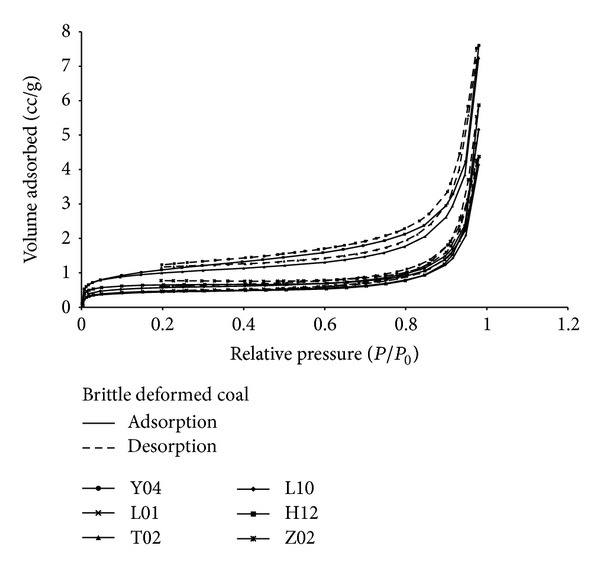
N_2_ gas adsorption/desorption isothermals of six brittle deformed coals.

**Figure 4:**
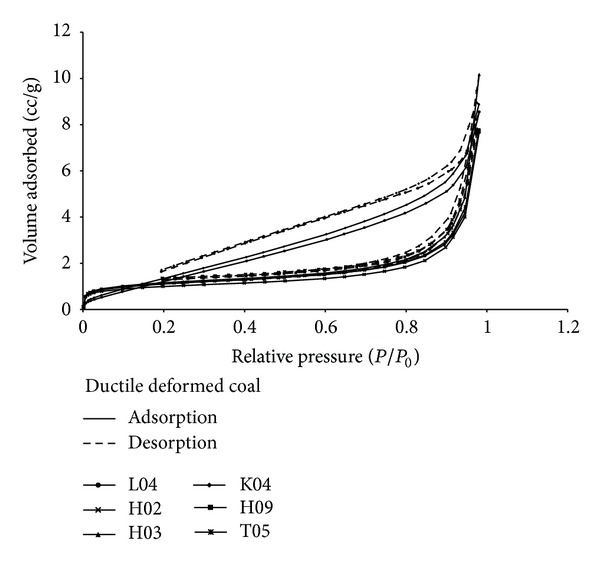
N_2_ gas adsorption/desorption isothermals of six ductile deformed coals.

The isotherms data for N_2_ gas adsorption at 77 K of brittle and ductile deformed coals are illustrated in Figures 3-4, respectively. According to the six adsorption/desorption isotherms of brittle deformed coal (Figure 3), the adsorption/desorption isothermals are reversible at the entire relative pressure range, which show the smallest deviations between adsorption and desorption curves (little hysteresis). The adsorption capacities of N_2_ in strong brittle deformed coal (H12 and T02) are much larger than that in weak brittle deformed coal at the same relative pressure.

The isothermals of ductile deformed coal can be divided into two types (Figure 4). One type shows little hysteresis (strong ductile deformed coal, H02, H09, H03, and T05), which is similar to the isothermals of brittle deformed coal. The other type exhibits a strong hysteresis at the entire relative pressure range (weak ductile deformed coal, L04, and K04). The adsorption capacities of N_2_ in weak ductile deformed coal (L04 and K04) are much larger than that in strong ductile deformed coal at relative pressure of 0.2–0.95. Furthermore, the adsorption capacities of N_2_ in ductile deformed coal are much larger than that in brittle deformed coal at the same relative pressure (Figures 3-4).

The shapes of adsorption/desorption isotherms reflect the pore shapes of coal [17, 27, 28]. There are two hysteresis types of 12 samples in this paper: types H3 (weak ductile deformed coal) and H4 (brittle deformed coal and strong ductile deformed coal) hystereses formed by slit shaped pores, with uniform (type H4) or nonuniform (type H3) size and/or shape.

### 3.3. Pore Volume (*V*) and Surface Area (*S*)

The parameters of the nanopores measured using a low-temperature nitrogen adsorption test, which include the pore width (*w*), the pore volume (*V*), and the surface area (*S*), are vital for the issues discussed in this paper.

**Figure 5:**
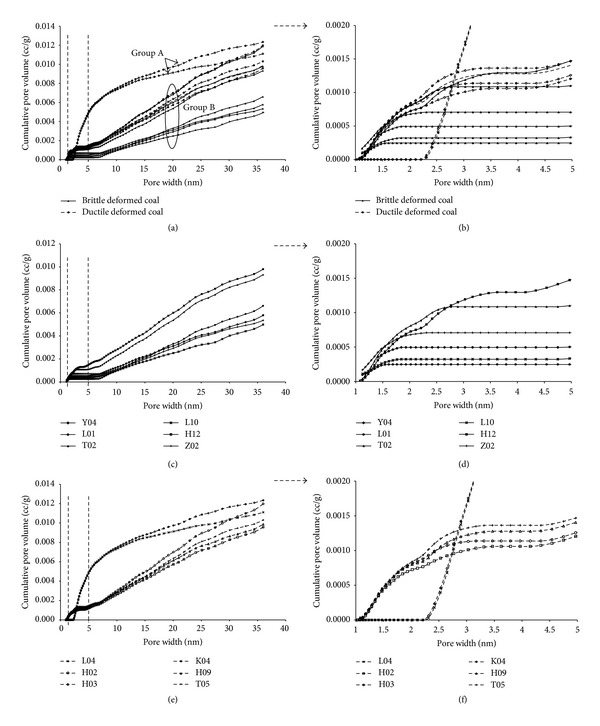
Relationship between cumulative pore volume and pore width of deformed coal (a-b); (c, d) brittle deformed coal; (e, f) ductile deformed coal.

The relationship between cumulative pore volume and pore width of deformed coal was shown in Figure 5. The cumulative pore volume of 12 samples had shown two groups: groups A and B (Figure 5(a)). The two lines shown at the top belong to group A whose pore volume increases significantly when the pore width is larger than 2 nm (Figure 5(b)). The others belong to group B whose pore volume increases fast at the beginning and shows large slope when the pore width increases (Figure 5(b)). For either brittle or ductile deformed coal, the larger the pore size is, the greater the pore volume becomes when the pore size is greater than 5 nm (Figure 5(a)).

Figures 5(c) and 5(d) show the cumulative pore volume lines of brittle deformed coal (belonging to group B). The four lines at the very bottom are weak brittle deformed coal whose pore volume increases fast when the pore size is greater than 7 nm. The two lines at the top are strong brittle deformed coal whose pore volume increases fast when the pore size is greater than 5 nm. Figure 5(c) also showed that the strong brittle deformed coals have greater pore volumes than that of weak brittle deformed coals. It means that, with the increase of the deformational intensity, the pore volume gradually increases in brittle deformed coal. The four lines at the bottom (group B) are strong ductile deformed coal, and the two lines at the top (group A) are weak ductile deformed coal which show large slope when the pore width is 2~10 nm (Figures 5(e) and 5(f)). The supermicropore (<2 nm) cannot be observed (Figure 5(f)) which means that there was almost no smaller pores in weak ductile deformed coal. The weak ductile deformed coals have greater volumes than that of strong ductile deformed coals (pore width range is 3~30 nm). It means that, with the increase of the deformational intensity, the pore volume gradually decreases in ductile deformed coal (Figure 5(e)).

**Figure 6:**
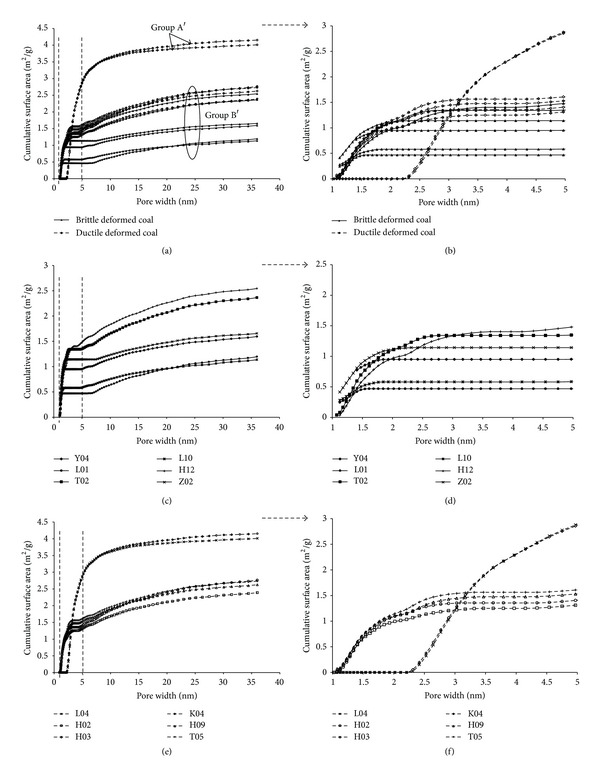
Relationship between cumulative surface area and pore width of deformed coal (a-b); (c, d) brittle deformed coal; (e, f) ductile deformed coal.

The cumulative surface area of 12 deformed coal samples (Figures 6(a) and 6(b)) also had shown two groups: groups A′ and B′. The two lines shown at the top belong to group A′ whose surface area increases significantly when the pore width is larger than 2 nm, especially in pore width range 2–5 nm (Figure 6(b)). The others belong to group B′, whose surface area increases fast at the beginning and shows small slope when the pore width increases. The smaller pores of coal (<5 nm) actually exhibit a greater surface area versus coal of larger pores (>5 nm) (Figure 6(a)).

The cumulative surface area lines of brittle deformed coal belong to group B′ (Figures 6(c) and 6(d)). The strong brittle deformed coals (the two lines at the top) have greater surface area than that of weak brittle deformed coals (the four lines at the bottom). It means that, with the increase of the deformational intensity, the surface area gradually increases in brittle deformed coal. The four lines at the bottom (group B′) are strong ductile deformed coal, and the two lines at the top (group A′) are weak ductile deformed coal; more than 60% surface areas are supplied by submicropores (2~5 nm) (Figures 5(e) and 5(f)). The weak ductile deformed coals have greater surface area than that of strong ductile deformed coals when pore width is greater than 3 nm.

We consider that the smaller pores significantly contribute to the surface area. However, the greater pores significantly contribute to the volume. The pore size in brittle deformed coal is greater than that in ductile deformed coal, but the cumulative pore volume and surface area are smaller than that in ductile deformed coal which indicates more adsorption space for gas. Compared with brittle deformed coal, there are more submicropores and supermicropores, but smaller quantities of mesopores and micropores in ductile deformed coal.

### 3.4. Distribution Characteristics of *S*-*V*

**Figure 7:**
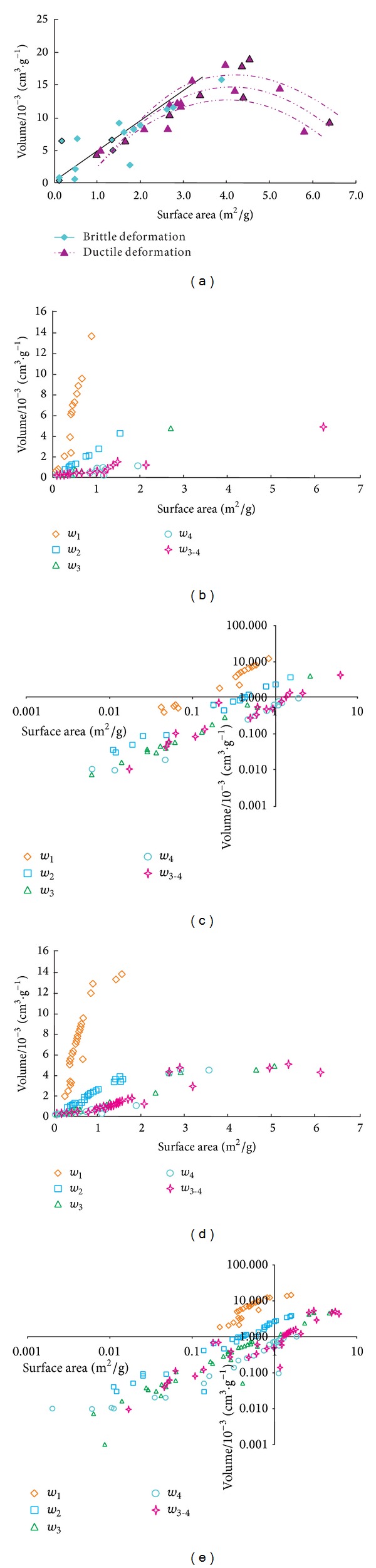
Relationship between *S* and *V* of deformed coal (a) in brittle and ductile deformed coal; (b, d) with different pore sizes in brittle and ductile deformed coals; (c, e) in log-log coordinate; *w*_1_–*w*_4_ mean different pore sizes; *w*_3-4_ means pore size which is less than 5 nm. The subscripts 1, 2, 3, and 4 are for mesopore (10~100 nm), micropore (5~10 nm), submicropore (2~5 nm), and supermicropore (<2 nm), respectively.

Figure 7(a) shows that there is a good linear relationship between *S* and *V* in brittle deformed coal but* V* first increases and then decreases with *S* increasing in ductile deformed coal. The relative trait of the two parameters of the pore structure exhibits a positive correlation when *S* is less than 4 m^2^/g. However, the relationship is broken when *S* is greater than 4 m^2^/g, as a negative correlation is exhibited.

The relationship between *S* and *V* with the different pore sizes in brittle and ductile deformed coal is shown in Figures 7(b), 7(c), 7(d), and 7(e), respectively. Figures 7(c) and 7(e) show the relationships between *S* and *V* in log-log coordinates. These figures indicate that the pore size has a close relationship with both *S* and *V*. Thus, larger pores exhibit the steepest slope, and smaller pores exhibit a low* S*-*V* slope in the two series of deformed coal. However, the negative correlation between *S* and* V*, which we mentioned before, is observed when the pore size is less than 5 nm in ductile deformed coal only. These results indicate that the *V* of smaller pore coal increased slowly with increasing* S*, and *S* of larger pore coal increased slowly with increasing *V*. In other words, a smaller pore size has a little influence on* V*, and a larger pore size has a little influence on* S*.

In ductile deformed coal,* V* decreases with increasing *S* when the pore size is less than 5 nm. The increase of *S* indicates that the number of smaller pores is increasing, and the number of larger pores is decreasing at the same time which reduces* V*, because the *V* of the increased number of smaller pores is much less than the *V* of the decreased larger pores. As a result,* S* and *V* will significantly increase and decrease, respectively, when the number of smaller pores reaches a certain amount.

### 3.5. Discussions

In raised temperature and confining pressure conditions, coal undergoes from low to high rank with the increase of temperature, when it has not been affected by tectonic stress. The aliphatic functional groups and branched n-alkane chains of the molecular structure of coal break off and gradually separate according to the bonding energy [1, 2, 21, 29–31]. Many types of hydrocarbons and nonhydrocarbons were formed. The molecules that are rearranged and concentrated through aromatization and polycondensation increase the degree of order and expand the number of basal structural unit (BSU) [1, 2, 16]. However, once the coal seams are influenced by tectonic stress, such stress will play an important role in the evolution of coal. Tectonic stress could affect and change the macromolecular structure in different ways, which cause the brittle and ductile deformation in a coal seam [21, 31, 32]. With the increase of deformational intensity, the brittle deformation can transform the stress into frictional heat energy and accelerate the movement of the small molecule. Such effects result in causing the aliphatic functional groups, alkane-branched chains, and the weak stability of CH in aromatic structure to be split away from the BSU [21, 29, 31]. Ductile deformation can transform the stress into strain energy through the accumulation of unit dislocations, and the strain rate of ductile deformation coal is slow, which provides a fraction of the cut small molecule enough time to form aromatic rings. Scholars considered that the changes of macromolecular structure could affect the nanopore structure (<100 nm), its distribution [2, 3], and various nanopore structures with good connectivity formed under different tectonic deformations and metamorphisms because of the irregular and dense array of the aromatic layer [6, 9, 31, 33].

**Figure 8:**
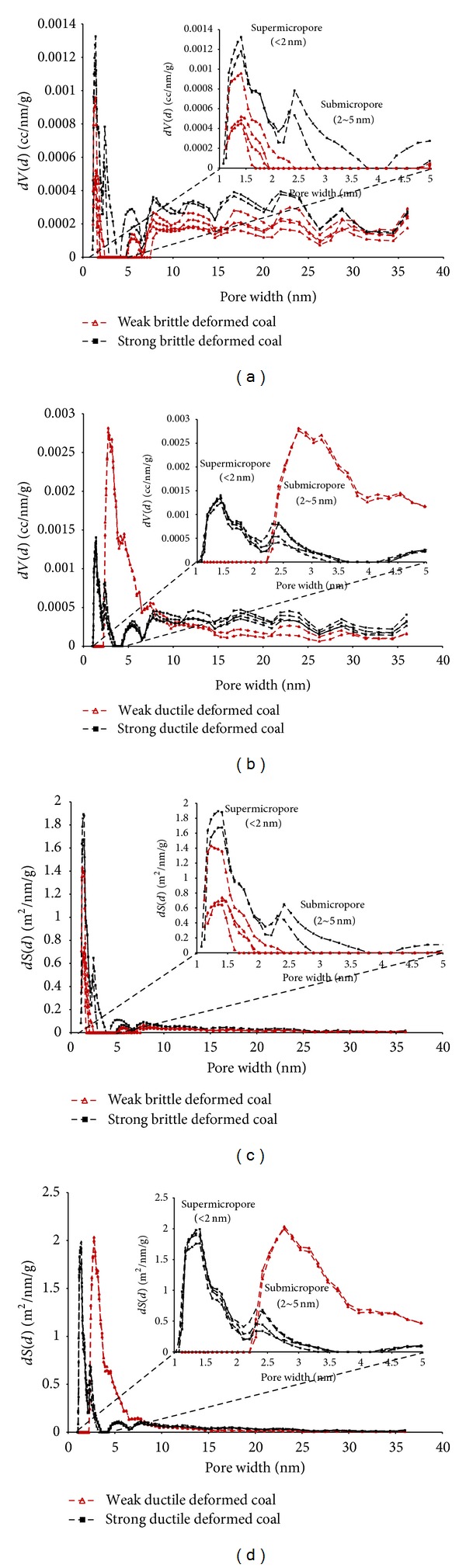
Nanopore distribution of deformed coals with different deformational intensity; (a, c) brittle deformed coal; (b, d) ductile deformed coal.

It is obvious that the quantity of pore (0–40 nm) in weak deformed coal is smaller than that in strong brittle deformed coal (Figures 8(a) and 8(c)). However, in weak ductile deformed coal, there are content of supermicropores (<2 nm) with little or no presence and a few pores with width greater than 5 nm (micropores (5~10 nm) and mesopores (10~100 nm)), but many submicropores (2~5 nm). All those submicropores in weak ductile deformed coal provide more than 35% volume and 60% surface area. With increasing deformational intensity, in strong ductile deformed coal, the quantity of supermicropores is obviously increased and the micropores and mesopores are increased too, while the submicropores are greatly reduced. It indicates that the tectonic stress could change the distribution of nanopore, and the interconversion will occur among the different size nanopores with increasing deformational intensity, especially in ductile deformed coal.

Zheng and Chen studied coal graphite using Raman spectroscopy which indicated that the process of tectonic stress and the lapping process were similar under the geological environment. Both of these processes could generate secondary structure defects, which can be observed by the peak value of* D* (related to the lattice vibration of irregular hexagon in amorphous graphite) [34, 35]. The Raman spectra of deformed coal were analyzed in our previous work [18, 21]. We found that the secondary structural defects are easier to generate and are present in a higher quantity in ductile deformed coal than in brittle deformed coal, because the secondary structural defect is a type of lattice defect, with a size equal to the size of a few benzene rings (0.7 nm). This observation indicates that a secondary structural defect has a size similar to that of a supermicropore or submicropore. With an increase of deformational intensity, secondary structural defects are generated in aromatic structures and aromatic layers through the dislocation glide process. These defects increase the number of submicropores and supermicropores.

**Figure 9:**
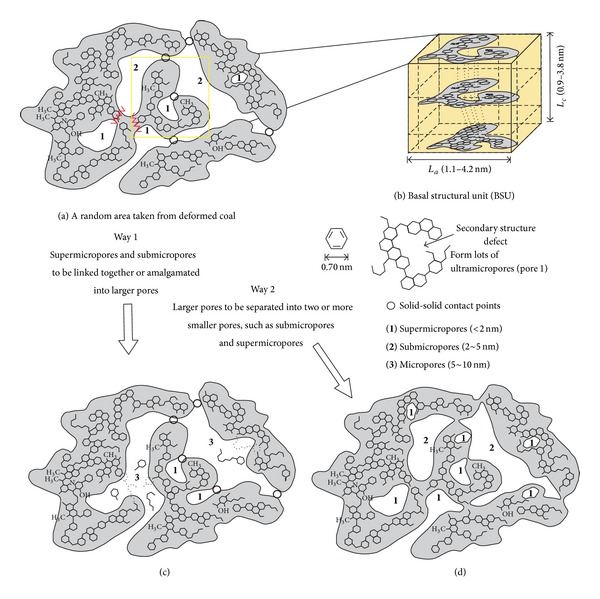
Evolutionary process of nanopore structure in deformed coal. (a) A random area taken from deformed coal. The black circle indicates a solid-solid contact point in deformed coal, number “1” means supermicropores, and number “2” means submicropores; (b) three-dimensional diagram of a small area which was magnified; (c) way 1 of interconversion among the different size nanopores: the parts of the smaller pores become connected or linked together and gradually grow into larger pores. Number “3” means micropores. (d) Way 2: larger pores being separated into two or more smaller pores.

Therefore, we consider that the macromolecular structure could change the nanopore structure (<100 nm) and its distribution in two ways (Figure 9). The first one is as follows (Figure 9(c)): a few secondary structural defects are generated. Meanwhile, aliphatic functional groups and branched n-alkane chains along the weak or unstable part are already broken off, inducing a connection between the supermicropores and the submicropores, which could cause parts of the supermicropores and submicropores to be linked together or amalgamated into larger pores (e.g., micropores). Yu et al. also found that most of the regular original small pores become connected and gradually grow into irregular and larger pores in coal [33]. The other way is as follows (Figure 9(d)): great quantities of secondary structural defects are generated, which will increase the number of submicropores and supermicropores. The small molecules are randomly spliced and embedded under polycondensation, inducing the larger pores to be separated into two or more smaller pores at the same time, such as submicropores and supermicropores.

## 4. Conclusions

(1) Compared with brittle deformed coal, there are more submicropores and supermicropores, but smaller quantities of mesopores and micropores in ductile deformed coal, which have greater pore volume and surface area and could provide more adsorption space for gas. The smaller the width is, the larger the value of surface area (*S*) is; the larger the width is, the greater the value of volume (*V*) is.

(2) There is a positive correlation between *S* and *V* in brittle deformed coal. However, in ductile deformed coal, the relationship between *S* and *V* turns from a positive correlation to a negative correlation when* S* > 4 m^2^/g for pore sizes < 5 nm. It is because that there are more submicropores and supermicropores, but smaller quantities of mesopores and micropores in ductile deformed coal; the *V* of the increased number of smaller pores is much less than the *V* of the decreased larger pores.

(3) The macromolecular structure could change the nanopore structure (<100 nm) and its distribution in two ways. The first one is as follows: the parts of the smaller pores become connected or linked together and gradually grow into larger pores (mesopore (10~100 nm), micropore (5~10 nm)). The other one is as follows: larger pores being separated into two or more small pores (submicropore (2~5 nm) and supermicropore (<2 nm)). Interconversion will occur among the different size nanopores especially in ductile deformed coal.

## Figures and Tables

**Table 1:** Vitrinite reflectance, maceral, and composition of deformed coal samples.

Deformation series	Sample ID	*R*_*o*,max⁡_/%	Deformation degrees	Maceral analysis/%			
				Vitrinite	Inertinite	Liptinite	Mineral
Brittle deformation	Y04	0.95	Weak	78.66	13.86	7.49	—
	L10	1.02	Weak	77.90	11.20	7.60	4.30
	L01	1.16	Weak	76.83	17.71	5.14	0.76
	H12	1.37	Strong	81.26	18.37	—	0.37
	T02	1.41	Strong	86.09	13.52	0.38	—
	Z02	1.93	Weak	84.03	14.82	—	1.14

Ductile deformation	L04	0.83	Weak	85.33	9.33	4.76	0.57
	K04	1.00	Weak	90.24	8.79	0.76	0.19
	H02	1.38	Strong	94.09	5.12	0.57	0.19
	H09	1.39	Strong	82.55	16.13	0.75	0.56
	H03	1.58	Strong	94.00	2.50	0.50	3.00
	T05	1.66	Strong	90.24	8.12	0.76	0.76

**Table 2:** DFT cumulative specific surface area, cumulative pore volume, and pore average width by N_2_ isotherm for deformed coal samples.

Deformation series	Sample ID	Deformation degrees	Specific surface area/m^2^ *·*g^−1^	Cumulative pore volume/cm^3^ *·*g^−1^	Average width/nm
Brittle deformation	Y04	Weak	1.5815	5.5416	16.3869
	L10	Weak	1.1240	4.7415	16.2702
	L01	Weak	1.1800	6.3164	20.5245
	H12	Strong	2.5297	9.5317	11.9679
	T02	Strong	2.3491	9.0517	12.6179
	Z02	Weak	1.6465	5.1731	12.5544

Ductile deformation	L04	Weak	4.0009	10.943	7.87924
	K04	Weak	4.1434	12.183	7.79493
	H02	Strong	2.3708	9.2548	13.4231
	H09	Strong	2.6091	9.5586	12.1140
	H03	Strong	2.7430	11.574	15.2008
	T05	Strong	2.7171	10.050	12.0523

**Table 3:** Classification of the nanopore structure of deformed coal.

Pore structure type	Distribution range of nanopore width/nm
Mesopore	10~100
Micropore	5~10
Submicropore	2~5
Supermicropore	<2
